# 

**Published:** 1916-07

**Authors:** 


### INDEX TO VOLUME XXXII.

ORIGINAL ARTICLES.

Page

The Diagnosis of the Internal Secretory Disorders. By Henry R. Harrower, M. D. (Article III continued)..... 7

Nervous Women and Gynecology. By F. S. White, M. D.. 9

New Fields of Medicine. By Dr. T. D. Crothers, Hartford, Conn........................................ 13

Summer Complaints Among Children. By R. C. Youngblood, M. D........................................ 19

The Diagnosis of the Internal Secretory Disorders (IV). By Henry R. Harrower, M. D., F. R. S. M. (Lond.), Los Angeles, California .................................. 47

The Great Yellow Peril. By Chas. H. Duncan, M. D., New York ................................................ 54

Paralysis Not Easily Caught. By Simon Flexner, M. D., New York ........................................ 59

Prevention of Infantile Paralysis—United States Public Health Service ........................................ 71

The Diagnosis of the Internal Secretory Diseases. By Henry R. Harrower, M. D., Los Angeles, California............ 95

Dementia Precox. By Dr. F. S. White, San Antonio, Texas. 99

The Great Yellow Peril—Medical Preparedness. By Chas. S. Duncan, M. D., New York.............................. 103

The Collection and Shipping of Pathological Specimens. By Dr. G. M. Graham, State Bacteriologist................ 108

The Diagnosis of the Internal Secretory Diseases. By Henry. R. Harrower, M. D., Los Angeles, California............ 143

The Great Yellow Peril. By Charles H. Duncan, M. D., New York City, N. Y........................................ 148

Treatment of Certain Types of Goitre With Quinine and Urea Injections. By L. F. Watson, M. D., Oklahoma City. 152

A Busy Doctor's Surgical Medley. By Dr. Neal Watt, Austin, Texas ........................................ 158

Heredity and Environment. By Dr. G. Henri Bogart, Shelbyville, Ill........................................ 161

Mouth Conditions as Related to the Physician's Work. By C. M. McCauley, B. S., D. D. S., Dallas, Texas.......... 163

Page

The Diagnosis of the Internal Secretory Disorders (IV). By Dr. Henry R. Harrower, Los Angeles, California........ 193
Texas State Health Board's Campaign for the Eradication of Malaria ........................................ 205
Mastication. By C. M. McCauley, B. S., D. D. S., Dallas, Texas ........................................ 205
The Treatment of Inoperable Cancer. By Dr. Silas P. Beebe, of New York................................ 207
Case of Foreign Body in the Urethra. By Dr. G. Frank Lydston, M. D., Chicago, Ill......................... 210
Delinquency from a Psychic Standpoint. By Dr. John S. Turner, Dallas, Texas.............................. 241
The Diagnosis of the Internal Secretory Disorders. By Henry R. Harrower, M. D., F. R. S. M. (Lond.), Los Angeles, California ........................................ 250
Observations of Blood Pressure as an Aid to Diagnosis. By Dr. Charles U. Patterson, Houston, Texas............. 257
Mouth Infections and Their Systemic Effect. By Arthur H. Merritt, D. D. S., New York......................... 262
The Rôle of Mouth Infection Abnormalities in the Causation of Diseases. By Dr. Eugene Lyman Fisk, New York.... 287
La Grippe, Its Symptomatology, Etiology and Treatment. By Dr. J. H. Wysong, Hico, Texas...................... 292
The Diagnosis of Internal Secretory Diseases. By Henry R. Harrower, M. D., Los Angeles, California.............. 296
Dental Caries. By C. M. McCauley, D. D. S., Dallas, Texas. 304
Iritis. By James C. Carter, M. D., Denison, Texas........ 311
Harrison Narcotic Law. By W. E. Allen, Assistant United States Attorney ........................................ 335
Obscure Symptoms of Syphilis and So-Called Parasyphilitic Diseases, With Discussion of Treatment. By I. T. McGlasson, M. D., Waco, Texas........................ 390
The Psychology of Charities. By T. D. Crothers, M. D.... 347
The Experience of Country Practice Necessary for a Complete Medical Training. By Dr. C. T. Bradford, Klondike, Texas ........................................ 351
The Etiology of Disease of the Bile-Bearing Passage. By Dr. C. O. Harper, Fort Worth, Texas................. 381
Gall Passage Diagnoses. By Dr. W. C. Calvert, Dallas, Texas ........................................ 384

Page

Medical Aspects of the Diseases of the Bile-Bearing Passages. By Dr. W. C. Morrow, Trenton, Texas................ 393
Tuberculin—Some Personal Experience. By Dr. W. O. Wilkes, Waco, Texas................................ 396
Recurrence After Thyroid Operation. By Dr. S. P. Beebe, New York ...................................... 403
A Plea for Complete Differential Blood Count in all Original Examinations. By Dr. C. L. Maxwell, Myra, Texas..... 413
What Does Pyorrhea Mean to the Work of the Physician? By Dr. C. M. McCauley, Dallas, Texas................. 419
A Method of Controlling Preventable Diseases in Rural Districts. By A. Caswell Ellis, Professor of the Philosophy of Education in the University of Texas.............. 429
The Pre-Cancerous Cervix. By S. A. Cunningham, M. D., San Antonio, Texas................................ 438
The Treatment of Extra-Uterine Pregnancy. By Aime Paul Heineck, M. D., Chicago, Ill., Professor of Surgery, Chicago College of Medicine and Surgery, Surgeon to Douglas, Jefferson Park and Francis Willard Hospitals........... 440
Salines in Hernias. By Dr. G. Henri Bogart, Shelbyville, Ill. ................................................ 452
Anoci-Association Applied in Obstretrics. By Dr. Malone Duggan, San Antonio, Texas........................ 477
The Use of Anesthetics and Pituitrin in Obstetrics. By Dr. L. H. Roddy, Waco, Texas.......................... 480
Pellagra: The Lack of Persistence in Its Treatment. By C. C. Parrish, M. D., Fort Worth, Texas............... 484
An Unusual Abdominal Tumor—Case Report. By Dr. M. Smith, Oklahoma City, Okla......................... 486
The Little Things in Obstetrics. By Dr. W. G. Harris, Plano, Texas ....................................... 488
Another Country Doctor Heard From.................... 490

### PELLAGRA FORUM.

Editorial. By Dr. E. H. Martin, Hot Springs, Ark........ 25
Pellagra. By H. C. Buck, M. D., Friars Point, Miss...... 26
The Cause of Pellagra and Treatment Suggestions. By B. W. Page, A. B., M. D.............................. 29
Editorial. By Dr. E. H. Martin, Hot Springs, Ark........ 73
Bread and Pellagra. By J. R. Lowery, M. D., Raleigh, N. C. 74

Page
Pellagra, Its Cause, Prevention and Cure. By E. M. Perdue, A. M., M. D., Kansas City, Mo ........ 78
Dr. Lee's Letter ........ 116
Pellagra Not a New Disease in America ........ 117
Dr. Van Zandt's Abstract ........ 118
Editor's Notes ........ 119
The Treatment of Pellagra. By Duane Meredith, M. D., Fort Worth, Texas ........ 167
Theories as to Pellagra ........ 213
The Colon, The Source of Infectious and Non-Infectious Systemic Diseases. By Dr. W. F. Cole, Waco, Texas ........ 215
Editorial. By E. H. Martin, Editor, Hot Springs, Ark ........ 269
The Editor's Chat ........ 273
Pellagra Department ........ 311

### RADIUM DEPARTMENT.

E. H. Skinner, M. D., Kansas City Mo., Editor.
J. M. Martin, M. D., Dallas, Assistant Editor.

Result and Present Position of Radium Therapy in Cancer of Uterus ........ 121
The Radium Fetich ........ 123
The Treatment of Leukoplakia by Radium. By Russell H. Boggs, M. D., Pittsburg, Pa ........ 224

### DEPARTMENT OF CLINICAL PATHOLOGY.

Dr. B. F. Stout, Editor, San Antonio, Texas ........ 222
Recent Reviews of Syphilis. By Dr. B. F. Stout, San Antonio, Texas ........ 312
Vaccine Treapy. By B. F. Stout, M. D ........ 355
Vaccination—A Review. By Dr. Theo. Hull ........ 357
Abstracts and Selections ........ 133, 134, 177, 233, 234, 323, 325, 328, 369, 370, 371, 372, 373, 374, 461, 511, 563
Items of General Interest. 37, 91, 175, 328, 369, 424, 460, 502, 559
Personals ........ 42, 461, 509, 563
Helpful Hints for the Busy Doctor ........ 44, 94, 140, 190, 236, 280, 330, 377, 427, 472, 518, 568
Book Reviews ........ 187, 279, 330, 566

### EDITORIAL DEPARTMENT.

Beginning a New Year ........ 33

To the New Subscriber ........ 34

Page
Dr. Martin's Pellagra Forum.............................. 35
Comments on the April Issue of the TEXAS MEDICAL JOURNAL ............................................ 36
Insanitary Surroundings ............................. 86
Autotheraphy ........................................ 88
Needless Pets ....................................... 90
An Impertinent Question............................. 90
Introducing the New Editors.......................... 125
The Physician's Opportunity.......................... 126
Dr. Sachs' Death..................................... 126
Mailing Poisons ..................................... 128
Increasing Circulation .............................. 129
Texas State Board of Heauth, Austin, Texas............. 130
Doing More Concrete Work............................. 131
Life's Mirror ....................................... 172
A Story of Fifty Years' Achievement.................. 172
The *Texas Medical News* to Be Enlarged............... 173
The Value of a Smile................................. 174
Reaching Out in Business............................. 227
Better Educated Physicians........................... 228
Read the Journals.................................... 229
Care of the Teeth.................................... 230
Army Medical Corps Examination....................... 230
Mineral Oil as an Intestinal Lubricant............... 231
A Christmas Prayer................................... 274
New Advertising ..................................... 274
The Christmas Season................................. 275
Battle Creek Sanitarium.............................. 277
Starch and Table Salt Sold as Neosalvarsan............ 278
Poem—Send It In...................................... 315
A New Feature........................................ 315
The New Year......................................... 316
Beautiful Sanitariums ............................... 317
Living Close to Nature............................... 318
Malaria and Money.................................... 319
Are We Germ Crazy?................................... 320
The Study of Internal Secretions..................... 321
Physical Training in Schools......................... 365
The Cancer Menace.................................... 366
Court Decision as to Vaccination..................... 367
Noticeable Improvement .............................. 368

Page
Poem ........................................ 422
Safeguarding the Medical School........................ 422
A Message from the Surgeon General to the Medical Profession ........................................ 424
Collections ........................................ 454
Medical Banquets ........................................ 455
A New Sanitarium in Austin........................ 456
The Doctor's Side of It (Poem)........................ 457
The Score (Poem) ........................................ 492
Fighting Malaria ........................................ 492
The Waste from Alcoholic Drinks........................ 493
Preventable Deaths ........................................ 493
After the War, What?........................ 494
Women and the War........................ 495
Our New Friends........................ 496
Dr. George H. Moody........................ 498
The Peril of the University of Texas........................ 551
An Indictment of the Whole Medical Profession—*American Medicine* ........................................ 552
Apologizing for Risque Stories........................ 555
A Menace Worse Than War........................ 555
Texas Training School for Defectives........................ 557
Salvarsan, an American Made Product........................ 558

### To the New Subscribers.

The TEXAS MEDICAL JOURNAL is privileged this month to extend a greeting and a welcome to nearly one thousand new subscribers. The rapidity with which the subscription list is increasing is indeed gratifying, and thanks are here given to all who have lately become readers of the JOURNAL. It is hoped and believed that you will be interested in every issue of the publication. If you read it closely you will be certain to find much that will be helpful. You have already noticed that the contributors are among the ablest men in the profession in this country, and that they write interestingly of what they know.

Please go carefully over every copy of the TEXAS MEDICAL

JOURNAL that you receive, and if you like it tell your fellow practitioners so. The time for professional jealousies has long since past, and every physician worthy of the name is anxious not only to learn all that he can, but also to help other physicians improve themselves. You can do this by increasing the circulation of the TEXAS MEDICAL JOURNAL. One Texas physician has personally sent in ten subscriptions from his fellow practitioners. He is not an agent of the JOURNAL and receives no commission, but is actuated solely by the good that he is doing.

Unfortunately, so many new subscriptions were received in June that the edition for that month was exhausted before all the orders were filled. Subscribers who failed to get the June number will receive this issue, and their subscriptions will date from July.

Editors are very human, and this editor will appreciate at all times any words of commendation or criticism from subscribers, and will be especially glad to have any suggestions for the improvement of the JOURNAL.

---

### Dr. Martin's Pellagra Forum.

### Deaths.

Dr. J. A. Root, of Granger, died recently.

Dr. Jim Nichols, of Bangs, died recently of typhoid fever.

Dr. A. G. Clopton died at the home of his daughter in Texarkana on June 20th.

Dr. Pink Montgomery, a prominent physician of Whitewright, was killed by a freight train recently as he was attempting to cross the track in his automobile on the Bonham and Whitewright road.

Dr. M. L. White, a resident of Austin for thirty-one years and prominently known in business circles, was found dead in bed at his camp at Deep Eddy on Friday morning, June 2d. An inquest held by Justice George Mendell gave the cause of his death as heart disease.

